# Racial differences in α4β7 expression on CD4^+^ T cells of HIV-negative men and women who inject drugs

**DOI:** 10.1371/journal.pone.0238234

**Published:** 2020-08-25

**Authors:** Alyssa R. Martin, Aida Sivro, Zoe R. Packman, Eshan U. Patel, Livia R. Goes, Lyle R. McKinnon, Jacquie Astemborski, Gregory D. Kirk, Shruti H. Mehta, Claudia Cicala, James Arthos, Andrew D. Redd, Thomas C. Quinn

**Affiliations:** 1 Division of Intramural Research, National Institute of Allergy and Infectious Diseases, National Institutes of Health, Bethesda, MD, United States of America; 2 Centre for the AIDS Programme of Research in South Africa (CAPRISA), Durban, South Africa; 3 Department of Medical Microbiology and Infectious Diseases, University of Manitoba, Winnipeg, Canada; 4 Department of Medical Microbiology, University of KwaZulu-Natal, Durban, South Africa; 5 Department of Pathology, Johns Hopkins University School of Medicine, Baltimore, MD, United States of America; 6 Department of Epidemiology, Johns Hopkins Bloomberg School of Public Health, Baltimore, MD, United States of America; 7 Department of Medicine, Johns Hopkins University School of Medicine, Baltimore, MD, United States of America; Ohio State University, UNITED STATES

## Abstract

**Introduction:**

We performed a cross-sectional study of HIV-uninfected men and women who inject drugs from the ALIVE cohort to examine if black men and women who inject drugs have higher levels of CD4^+^ T cells expressing the integrin heterodimer α4β7 compared to white men and women.

**Materials and methods:**

Flow cytometry was used to examine expression of α4β7 and other markers associated with different functional CD4^+^ T cell subsets in both men and women who inject drugs.

**Results:**

Higher levels of α4β7, CCR5, and CCR6 were observed on CD4^+^ T cells from black participants compared with white participants. In a multivariable model, α4β7 expression differed by race, but not sex, age, or other factors.

**Discussion:**

Black men and women express higher percentages of α4β7 expressing CD4^+^ T cells, which may play a role in HIV disease.

## Introduction

α4β7, a gut-homing integrin heterodimer expressed on CD4^+^ T cells, may play a role in HIV acquisition and disease progression. CD4^+^ T cells expressing high levels of α4β7 are most likely to become infected with HIV *in vitro* [1], with simian immunodeficiency virus (SIV) in vivo [2], and are specifically depleted during acute HIV-1 infection [3,4]. In a non-human primate model for HIV infection, *in vivo* administration of an monoclonal antibody to α4β7 reduced mucosal transmission of SIV in macaques [5], and was initially shown to possibly confer virologic control in monkeys previously infected with SIV [6]. However, this latter finding was not reproduced in subsequent primate studies [7]. In humans, a higher frequency of α4β7^hi^ CD4^+^ T cells has been associated with an increased risk of HIV acquisition and more rapid disease progression in heterosexually infected African women [4].

Given these previous studies, it is possible that certain factors, genetic or immunologic, that affect α4β7 expression may alter an individual’s risk of HIV infection. A study of HIV-negative men who have sex with men (MSM) in the United States suggested that there may be a racial difference in α4β7 expression on CD4^+^ T cells, with black MSM having higher frequencies of α4β7^hi^ CD4^+^ T cells than their white MSM counterparts [8]. It is possible that this difference in α4β7 expression may contribute to previously unexplained racial disparities in HIV acquisition [9]; however, this racial difference remains to be confirmed in men and has not been examined in women. Previous evidence has also suggested that HSV-2 infection may increase HIV infection risk in part by upregulation of α4β7 on CD4^+^ T cells [10]. Of note, HSV-2 infection has been associated with significantly increased α4β7 expression on blood T cells from a cohort of African/Caribbean women [11].

Cellular differentiation may also influence expression of α4β7. The regulatory subset of CD4^+^ T cells (Tregs) are a component of gut-associated lymphoid tissue (GALT) and have the potential to express high levels of α4β7 [3]. Th17 cells, which express CCR6, and are overrepresented in the GALT, are also highly susceptible to infection. It is important to know if these cells, as well as CCR5^+^ and activated T cell subsets, may also express increased levels of α4β7 compared to the total CD4^+^ T cell population [1,12,13]; and if these differences in numbers and/or proportions of these specific subsets may contribute to the difference in α4β7 expression by race. In this study, we sought to examine the association of race and α4β7 expression in HIV-negative people who inject drugs and explore other factors that may influence this association.

## Methods

### Study population

We tested a total of 110 PBMC samples from 100 HIV-uninfected individuals including 25 white men, 25 white women, 25 black men, and 25 black women enrolled in the AIDS Linked to the IntraVenous Experience (ALIVE) study in Baltimore, MD. Participant samples were selected based on sex, race, and sample collection within 3 years of study initiation. The protocol requirements for the ALIVE study have been previously described [14]. All participants were ≥18 years of age or older, had a history of injection drug use and provided informed consent. This study was approved by the Johns Hopkins University Institutional Review Board. For a subset of 10 individuals, two samples collected six months to two years apart were tested to measure stability of α4β7 expression over time. Active drug use was self-reported at time of sample collection. Two samples were of insufficient quality and were therefore excluded from analysis.

### Quantification of cellular factors

Flow cytometry was used to measure the expression of cell markers. Quantification of α4β7 levels was performed by gating β7^+^/CD45RA^-^ within the population of CD4^+^ T cells to identify β7^hi^ populations based on previously published methods and data [1,4]. Two panels of fluorescently labeled antibodies were used to characterize each sample. An aliquot of each sample was stained using the following antibodies: PE-Cy5-HLA-DR_555813, FITC-CD38_555459, PE-Cy7-CD27_560609, PE-CF594-CCR7_562381, BV711-CD45RA_563733, BV421-CCR5_562576, PE-Integrin β7_555945, BV650-CD8_563821, APC-H7-CD3_560176, BV605-CD4_562658, AlexaFluor 647-Ki67. An additional aliquot was stained with a panel of antibodies to assess additional T cell phenotypes: APC-H7-CD3_560176, BV421-CD25_564033, PE-CF594-CD39_563678, BV711-CD127_563165, BV786-CD152/CTLA4_563931, PE-FoxP3_12-4777-42 (Thermo Fisher), PE-Cy5-Integrin β7_551059, BV605-CD4_562658, FITC-CD45RA_555488, BV650-CCR6_563922. All antibodies were purchased from BD Biosciences unless otherwise indicated. Aliquots were also stained with LIVE/DEAD Fixable Aqua Dead Cell Stain Kit, (Invitrogen). Th17 cells were estimated by examining CCR6+ CD4+ cell populations. Tregs were defined as CD25^+^CD127^-^ or by CD25^+^FoxP3^+^; no significant difference was seen between these gating strategies (S3 Fig). Flowjo software was used to analyze flow cytometry data.

### Assessment of Herpes Simplex Virus-2 (HSV-2) and Hepatitis C virus (HCV) status

HSV-2 serostatus was determined by ELISA (Kalon Biological, England) on serum or plasma samples according to the manufacturers’ recommendation. Serum or plasma was unavailable for seven participants, and results were inconclusive for two samples; these participants were excluded from this portion of the analysis. HCV serostatus was determined by ELISA (Ortho HCV Version 3.0. Raritan, NJ) on serum samples.

### Statistical analysis

Characteristics of the study population were examined using descriptive statistics. Fisher’s exact, chi-square tests and Mann-Whitney tests were performed to examine racial differences in the distribution of categorical and continuous variables, respectively. Demographic, risk, and inflammation-related factors associated with % CD4^+^β7^hi^ expression were individually examined using linear regression. Variables shown to be associated with α4β7 expression in univariable analysis (p<0.05) and those deemed important a priori (i.e., age, sex, and race), regardless of statistical significance, were included in a multivariable linear regression model. A two-sided p-value less than 0.05 was considered statistically significant. Stata software package was used for linear regression analyses, and Graphpad Prism were used for all other analyses.

## Results

Samples were collected from individuals seronegative for HIV with a history of injection drug use (n = 98; Table 1). 38.8% of participants reported injection drug use in the past six months, and 74.5% were seropositive for HCV. Of the 88 individuals that were analyzed, 51 (58.0%) were HSV-2 seropositive. Two flow cytometry panels were used to determine cellular factors associated with differential β7^i^ expression. There was little variation in β7 levels when compared between the two flow panels that were used for each sample (r = 0.944, p<0.0001; S1A and S1B Fig). In 10 patients that were sampled at two time points up to two years apart, we found that % CD4^+^ β7^hi^ expression was stable for up to two years, with a median coefficient of variation of 5% (S1C Fig).

**Table 1:** Characteristics of the study population.

Characteristic	No. of Participants (%)			*P* value
	Overall (n = 98)	Black race (n = 49)	White race (n = 49)	
Age (years), median (IQR)	50 (38–60)	61 (57–64)	38 (31–44)	**<0.001**
Sex				1.000
Male	49 (50.0%)	25 (51.0%)	24 (49.0%)	
Female	49 (50.0%)	24 (49.0%)	25 (51.0%)	
Race				-
Black	49 (50.0%)	-	-	
White	49 (50.0%)	-	-	
Injection drug use in past 6 mo.				**<0.001**
No	60 (61.2%)	43 (87.8%)	17 (34.7%)	
Yes	38 (38.8%)	6 (12.2%)	32 (65.3%)	
HCV IgG antibody status				**<0.001**
Seropositive	73 (74.5%)	45 (91.8%)	28 (57.1%)	
Seronegative	25 (25.5%)	4 (8.2%)	21 (42.9%)	
HSV-2 IgG antibody status				**<0.001**
Seropositive	51 (52.0%)	35 (71.4%)	16 (32.7%)	
Seronegative	37 (37.8%)	12 (24.5%)	25 (51.0%)	
*Missing*	10 (10.2%)	2 (4.1%)	8 (16.3%)	
BMI, median (IQR)	27.8 (23.1–31.2)	27.5 (23.5–31.2)	28.1 (22.7–31.2)	0.986

Data are the sample sizes and corresponding column percentages, unless otherwise indicated. To compare characteristics by race, *P* values were calculated from Mann-Whitney tests and Fisher’s exact tests for continuous and categorical variables, respectively. Abbreviations: HCV, hepatitis C virus; HSV-2, herpes simplex virus type 2; BMI, body mass index; IQR, interquartile range.

**Fig 1:**
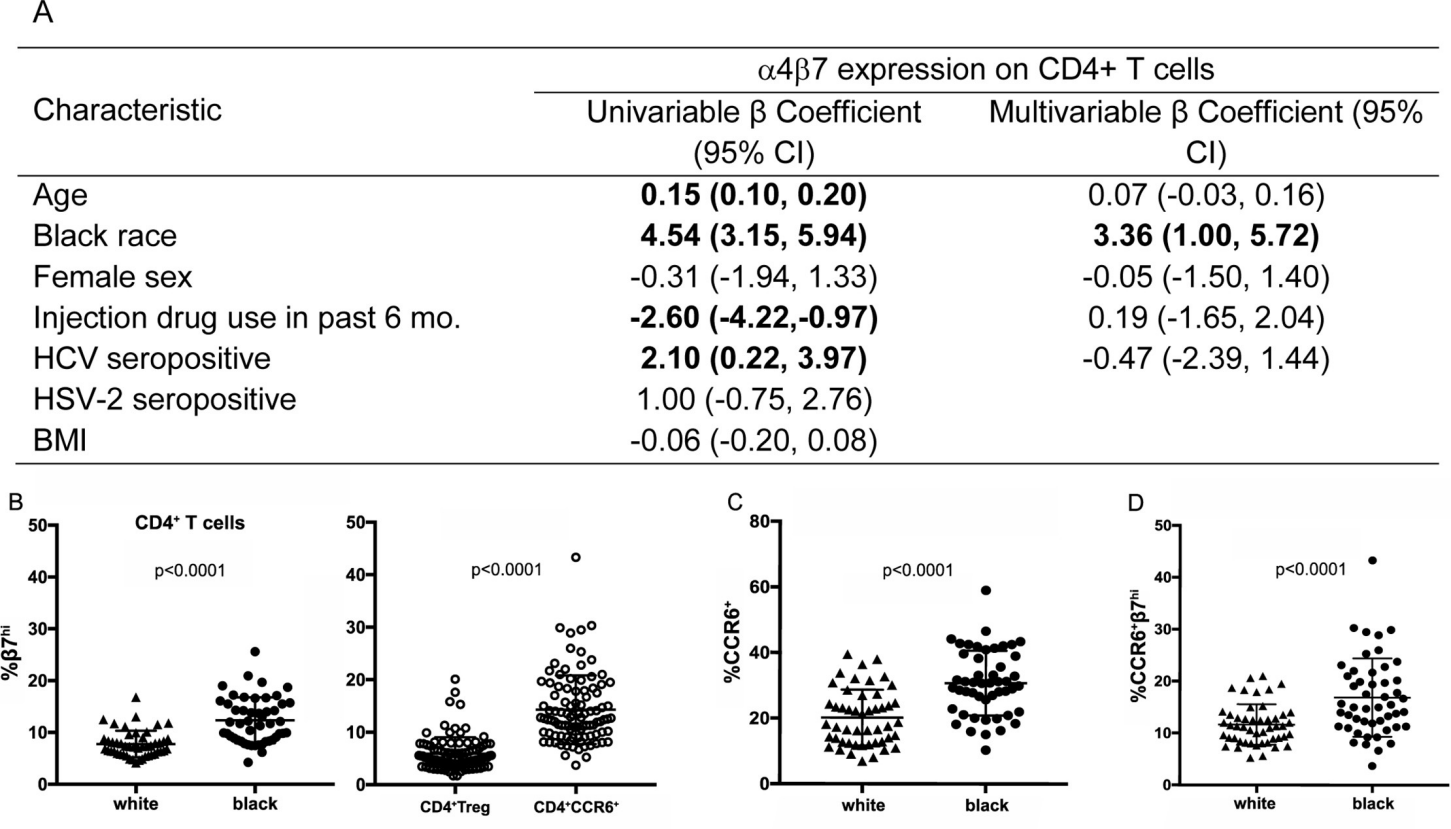
Measurement of α4β7 expression on total CD4^+^ T cells and subsets, and differences by race. A) Correlates of α4β7 expression on CD4+ T cells examined using linear regression. Multivariable analysis included age, black race, sex, injection drug use status, and HCV status. Only race varied in β7 expression in multivariable analysis. B) β7^hi^ expression on CD4^+^ T cells by race and T cell subset. White participants had significantly lower proportions of β7^hi^ CD4^+^ T cells than black participants. CD4^+^ CCR6^+^ cells had higher % β7^hi^ than Tregs. C) Differences in proportion of CD4^+^ CCR6^+^ cells by race. Black individuals had overall higher proportions of CD4^+^CCR6^+^ cells than white participants. D) Racial differences in % CD4^+^ β7^hi^CCR6^+^ cells by race. Crossbars represent the mean and standard deviation.

In univariable linear regression analysis, older age, black race, and HCV-positive serostatus were factors significantly associated with higher levels of % CD4^+^ β7^hi^ expression, whereas injection drug use in the past six months was significantly associated with lower levels of % CD4^+^ β7^hi^ expression (p<0.05). Sex, HSV-2 serostatus, and BMI were not significantly associated with % CD4^+^ β7^hi^ expression. In the multivariable model that included, age, race, sex, injection drug use in the past six months, and HCV serostatus, the only factor that remained significantly associated with % CD4^+^ β7^hi^ expression was black race (β = 3.36, 95%CI: 1.00–5.72; p<0.05; Fig 1A). This measurement of higher proportions of % CD4^+^ β7^hi^ cells in black individuals is in accordance with previously reported data [8]. However, β7 expression was not significantly different by race as measured by mean fluorescence intensity (MFI) (p = 0.4 S2A Fig).

We next determined whether any specific cell types were associated with high α4β7 expression. To establish that the observed racial difference was not simply associated with an overall difference in immune activation, we measured β7 expression specifically on activated (CD38^+^HLA-DR^+^) cells. Although these activated cells did in fact contain a higher proportion of β7^hi^ cells compared to non-activated (S2B Fig), there was no difference in the proportion of activated cells by race, consistent with previously reported data [8] (S2C Fig). In contrast, CCR5^+^ cells were present at higher proportions in black individuals in our study (S2D Fig). These cells did express higher levels of β7 than CCR5^-^ cells, likely due to differences in activation status; however, we did not find a higher proportion of CCR5^+^β7^hi^ cells by race (S2E Fig). Together these results suggest that activation status of CD4^+^ T cells did not account for the overall β7^hi^ expression differences by race.

However, differences in helper T cell subsets may have had a role in racial differences in β7^hi^ expression. CCR6^+^ cells were more likely to be β7^hi^ compared with Tregs (Fig 1B). The proportion of CCR6^+^ cells varied by race (Fig 1C) and black individuals had higher CCR6^+^β7^hi^ expression (Fig 1D), indicating that the CCR6^+^ subset of CD4^+^ may have contributed to the overall β7^hi^ expression differences by race.

## Discussion

HIV incidence has been shown to be higher in black men and women when compared to their white counterparts, and is not entirely explained by a disparity in behavioral risk factors [9]. It was previously found that higher expression of α4β7 on CD4^+^ T cells was associated with increased risk of HIV acquisition in African heterosexual women [4]. In addition, racial differences in α4β7 expression on CD4^+^ T cells have been found in HIV-uninfected MSM, with black men having higher proportions of α4β7 expressing CD4^+^ T cells [8]. Taken together, these findings suggest that it is possible that increased α4β7 on CD4^+^ T cells may contribute to an increased risk of HIV infection for black men and women.

In the present study, we report that α4β7 expression on CD4^+^ T cells was elevated in the black participants in the ALIVE cohort, which is based in Baltimore, Maryland. It should be noted that due in large part to the drug use patterns in different populations participating in the cohort (black participants tend to be older compared to white people who inject drugs), our study population included several differences in other risk/demographic factors by race, which in a univariable model were associated with α4β7 expression. However, a multivariable analysis indicated that the strongest, and only significant, association with α4β7 expression was race, but our study may have lacked the power to query some of these other factors such as age. Additionally, it is possible that residual confounding may exist This also may explain why no difference in α4β7 expression by HSV-2 status was observed in this population, which is inconsistent with a previous study that observed HSV-2 seropositive women were more likely to have increased α4β7 on CD4^+^ T cells [11]. Another limitation of the study is that there is no way to know how mixed-race individuals would self-identify when they were recruited for the study.

We also examined α4β7 expression on several T cell subsets to further examine this phenomenon, and found expression to be elevated on CCR6^+^ T cells. CCR6 is a marker used to identify Th17 cells, which are overrepresented in the GALT, and are among the major cellular targets for HIV being highly susceptible to infection [15]. Previous studies have also demonstrated that both CD4^+^CCR6^+^ T cells and α4β7 expressing cells are specifically depleted during HIV infection [3,12]. Taken together, this might suggest that it is possible that the higher α4β7 expression observed on CCR6+ cells in black participants may contribute to possible increased risk of infection and disease progression in these individuals. However, the role of α4β7 expressing cells in HIV cure, disease progression, and acquisition is not fully understood with conflicting *in vitro*, epidemiological, and non-human primate findings, especially in regards to cure research. Therefore, further research is needed to fully understand the role these cells may play in HIV acquisition and disease progression.

## Supporting information

S1 Fig CD4^+^β7^hi^ expression over time and by sex and race. A. Gating strategies used to determine CD4^+^β7^hi^ expression. Two flow cytometry panels were used to determine cellular factors associated with differential β7^hi^ expression. B. Correlation of β7^hi^ levels as measured between two flow panels run on each sample. C. Stability of CD4^+^β7^hi^ expression. Patients (n = 10) were sampled twice between a 6 month to 2-year period to determine variance of β7^hi^ expression over time.
(DOCX)

S2 Fig α4β7 expression and racial differences in activated CD4^+^ cells. A. β7^hi^ mean fluorescence intensity (MFI) by race. No difference in MFI was measured between white and black participants. B. β7^hi^ levels vary by cellular activation levels. CD4^+^ CD38^+^HLA-DR^+^ cells are more likely to be β7^hi^ than CD4^+^ CD38^-^HLA-DR^-^. C. Cellular activation levels by race. No difference was measured in proportion of CD4^+^ CD38^+^HLA-DR^+^ cells between white and black populations. D. Racial differences in CD4^+^CCR5^+^ cells. A greater proportion of CD4^+^ T cells were CCR5^+^ in black participants versus white. E. Differences in β7^hi^ expression by race in CD4^+^CCR5^+^ cells. Though CD4^+^ CCR5^+^ cells had significantly higher %β7^hi^ than CD4^+^ CCR5^-^, there was no difference in CD4^+^ CCR5^+^β7^hi^ expression by race. Crossbars represent the mean and standard deviation.
(DOCX)

S3 Fig Flow cytometry gating used to determine regulatory T cell (Treg) percentages. No significant difference was seen between cells identified as Tregs between strategies. Representative data from one sample is shown. In addition, to verify this population the parent population was gated into the second group to measure the direct overlap. When we gated strategy 1 within strategy 2, they agreed 76.5% (SD = 7.3).
(DOCX)
