# Supplementary material for: Racial differences in α4β7 expression on CD4+ T cells of HIV-negative men and women who inject drugs
Source: PLoS One. 2020 Aug 25;15(8):e0238234. doi: 10.1371/journal.pone.0238234 (PMC7447027; doi:10.1371/journal.pone.0238234)
Supplement: S1 Fig — A. Gating strategies used to determine CD4+β7hi expression. Two flow cytometry panels were used to determine cellular factors associated with differential β7hi expression. B. Correlation of β7hi levels as measured between two flow panels run on each sample. C. Stability of CD4+β7hi expression. Patients (n = 10) were sampled twice between a 6 month to 2-year period to determine variance of β7hi expression over time. (DOCX) [file pone.0238234.s001.docx]

**S1 Fig**. CD4^+^β7^hi^ expression over time and by sex and race.

A. Gating strategies used to determine CD4^+^β7^hi^ expression. Two flow cytometry panels were used to determine cellular factors associated with differential β7^hi^ expression.

B. Correlation of β7^hi^ levels as measured between two flow panels run on each sample.

C. Stability of CD4^+^β7^hi^ expression. Patients (n = 10) were sampled twice between a 6 month to 2-year period to determine variance of β7^hi^ expression over time.
