# Supplementary material for: Racial differences in α4β7 expression on CD4+ T cells of HIV-negative men and women who inject drugs
Source: PLoS One. 2020 Aug 25;15(8):e0238234. doi: 10.1371/journal.pone.0238234 (PMC7447027; doi:10.1371/journal.pone.0238234)
Supplement: S2 Fig — A. β7hi mean fluorescence intensity (MFI) by race. No difference in MFI was measured between white and black participants. B. β7hi levels vary by cellular activation levels. CD4+ CD38+HLA-DR+ cells are more likely to be β7hi than CD4+ CD38-HLA-DR-. C. Cellular activation levels by race. No difference was measured in proportion of CD4+ CD38+HLA-DR+ cells between white and black populations. D. Racial differences in CD4+CCR5+ cells. A greater proportion of CD4+ T cells were CCR5+ in black participants versus white. E. Differences in β7hi expression by race in CD4+CCR5+ cells. Though CD4+ CCR5+ cells had significantly higher %β7hi than CD4+ CCR5-, there was no difference in CD4+ CCR5+β7hi expression by race. Crossbars represent the mean and standard deviation. (DOCX) [file pone.0238234.s002.docx]

**S2 Fig**. α4β7 expression and racial differences in activated CD4^+^ cells.

A. β7^hi^ mean fluorescence intensity (MFI) by race. No difference in MFI was measured between white and black participants. B. β7^hi^ levels vary by cellular activation levels. CD4^+^ CD38^+^HLA-DR^+^ cells are more likely to be β7^hi^ than CD4^+^ CD38^-^HLA-DR^-^. C. Cellular activation levels by race. No difference was measured in proportion of CD4^+^ CD38^+^HLA-DR^+^ cells between white and black populations. D. Racial differences in CD4^+^CCR5^+^ cells. A greater proportion of CD4^+^ T cells were CCR5^+^ in black participants versus white. E. Differences in β7^hi^ expression by race in CD4^+^CCR5^+^ cells. Though CD4^+^ CCR5^+^ cells had significantly higher %β7^hi^ than CD4^+^ CCR5^-^, there was no difference in CD4^+^ CCR5^+^β7^hi^ expression by race. Crossbars represent the mean and standard deviation.
