# Supplementary material for: Racial differences in α4β7 expression on CD4+ T cells of HIV-negative men and women who inject drugs
Source: PLoS One. 2020 Aug 25;15(8):e0238234. doi: 10.1371/journal.pone.0238234 (PMC7447027; doi:10.1371/journal.pone.0238234)
Supplement: S3 Fig — No significant difference was seen between cells identified as Tregs between strategies. Representative data from one sample is shown. In addition, to verify this population the parent population was gated into the second group to measure the direct overlap. When we gated strategy 1 within strategy 2, they agreed 76.5% (SD = 7.3). (DOCX) [file pone.0238234.s003.docx]

**S3 Fig**. Flow cytometry gating used to determine regulatory T cell (Treg) percentages. No significant difference was seen between cells identified as Tregs between strategies. Representative data from one sample is shown. In addition, to verify this population the parent population was gated into the second group to measure the direct overlap. When we gated strategy 1 within strategy 2, they agreed 76.5% (SD=7.3).
